# The Pandemic Threat of Emerging H5 and H7 Avian Influenza Viruses

**DOI:** 10.3390/v10090461

**Published:** 2018-08-28

**Authors:** Troy C. Sutton

**Affiliations:** 1 Department of Veterinary and Biomedical Sciences, Center for Molecular Immunology and Infectious Disease, The Pennsylvania State University, University Park, PA 16802, USA; tcs38@psu.edu; Tel.: +1-814-863-0693; 2 The Huck Institutes of Life Sciences, Center for Infectious Disease Dynamics, The Pennsylvania State University, University Park, PA 16802, USA

**Keywords:** influenza, pandemic, airborne transmission, highly pathogenic avian influenza

## Abstract

The 1918 H1N1 Spanish Influenza pandemic was the most severe pandemic in modern history. Unlike more recent pandemics, most of the 1918 H1N1 virus’ genome was derived directly from an avian influenza virus. Recent avian-origin H5 A/goose/Guangdong/1/1996 (GsGd) and Asian H7N9 viruses have caused several hundred human infections with high mortality rates. While these viruses have not spread beyond infected individuals, if they evolve the ability to transmit efficiently from person-to-person, specifically via the airborne route, they will initiate a pandemic. Therefore, this review examines H5 GsGd and Asian H7N9 viruses that have caused recent zoonotic infections with a focus on viral properties that support airborne transmission. Several GsGd H5 and Asian H7N9 viruses display molecular changes that potentiate transmission and/or exhibit ability for limited transmission between ferrets. However, the hemagglutinin of these viruses is unstable; this likely represents the most significant obstacle to the emergence of a virus capable of efficient airborne transmission. Given the global disease burden of an influenza pandemic, continued surveillance and pandemic preparedness efforts against H5 GsGd and Asian lineage H7N9 viruses are warranted.

## 1. Introduction

### 1.1. Avian Origin of 1918 H1N1 “Spanish Influenza” Pandemic

This year marks 100 years since the 1918 H1N1 “Spanish Influenza” pandemic. This was the most severe pandemic in modern history and is estimated to have infected 500 million people worldwide (one-third of the global population) [1], resulting in 50–100 million deaths [2,3]. Early comparison of the 1918 H1N1 viral RNA sequences indicated that the virus crossed directly from birds into humans [4,5,6]. However, this analysis was controversial due to limited contemporary avian influenza gene sequences and conflicting phylogenic analyses [7,8,9]. Recent analysis using a host-specific molecular clock to evaluate the evolution of the 1918 H1N1 virus strongly indicates that the polymerase basic 2 (PB2), polymerase basic 1 (PB1), polymerase acidic (PA), nucleoprotein (NP), neuraminidase (NA), matrix (M), and non-structural (NS) gene segments were derived directly from an avian host, while the H1 hemagglutinin (HA) may have been contributed via reassortment with a human virus that emerged between 1915 and 1917 [10,11]. Thus, while more recent influenza pandemics emerged via reassortment of multiple gene segments, the 1918 H1N1 virus is unique as most of the genes were derived directly from an avian influenza virus. Moreover, as all subsequent pandemic influenza viruses have contained gene segments derived from the 1918 H1N1 virus, this virus has earned the designation as the “mother” of all pandemics [1,12].

Influenza A viruses are subtyped based on their combination of HA and NA surface glycoproteins, and there are 18 HA and 11 NA subtypes [13,14,15,16]. Sixteen HA subtypes and nine NA subtypes circulate in wild aquatic birds, which are considered the natural reservoir for influenza A viruses [13,14,15]. Two subtypes, H17N10 and H18N11, have been identified in bats using viral RNA sequence analysis [16]. After the 1918 pandemic, H1N1 viruses continued to circulate in humans and these viruses displayed reduced morbidity and mortality. In 1957 an H2N2 virus emerged and initiated a pandemic. This virus replaced the circulating H1N1 viruses and circulated until 1968, when it was replaced by a pandemic H3N2 virus [17]. In 1977, an H1N1 virus again initiated a pandemic. This H1N1 virus originated in China or Russia and shared high sequence identity with the H1N1 viruses that circulated prior to 1957 [18,19,20]. Due to this high degree of similarity and a lack of mutations that would indicate circulation in another host, the reemergence of these viruses has been attributed to an accidental release or failed vaccine trial [21]. In contrast to the previous influenza pandemics, the 1977 H1N1 viruses did not replace the circulating H3N2 viruses, and both the H3N2 and H1N1 viruses began to co-circulate in humans. When the 2009 pandemic H1N1 virus emerged, this virus replaced the circulating H1N1 viruses, but again H3N2 viruses continued to circulate. With the exception of the 1977 H1N1 viruses, all pandemic viruses since 1918 have been reassortant viruses that derived the viral HA from avian (i.e., H2 and H3) or swine (i.e., 2009 H1N1) influenza viruses [17]. Humans, therefore, had little or no pre-existing immunity to these viruses, which allowed the viruses to spread rapidly in the population. In recent years, avian viruses of the H5, H7, H9, and H10 subtypes have caused zoonotic infections with H5 and H7 viruses often causing severe disease. As a result, there is significant concern that viruses from these subtypes could evolve and initiate the next pandemic.

### 1.2. Properties of Pandemic Influenza Viruses

To start a pandemic, an influenza virus must overcome two major obstacles. First, the virus must be antigenically novel to humans, and secondly, the virus must be able to spread efficiently from person-to-person. Humans have no pre-existing immunity to H5 or H7 avian influenza viruses. Thus, if an H5 or H7 virus is introduced into the human population and can spread between people, particularly via the airborne route, it will initiate a pandemic. With this in mind, both the Centers for Disease Control (CDC) and the World Health Organization (WHO) use airborne transmission (also referred to as respiratory droplet transmission) between experimental animals to assess the pandemic potential or risk posed by an emerging influenza virus [22,23].

Experimental animals used to assess and model airborne transmission in humans include ferrets and guinea pigs [24,25]. Ferrets are the preferred model because they exhibit clinical signs upon influenza infection [26], have similar cell surface receptor (i.e., sialic acid) distribution in the respiratory tract to humans [27,28,29], and efficiently transmit (i.e., transmission to 75–100% of contacts) pandemic influenza viruses via direct contact and respiratory droplets [30,31,32,33]. Transmission studies in guinea pigs have largely reproduced the findings in ferrets, and due to the reduced cost and ease of handling, guinea pigs can be utilized in larger numbers [25]; however, unlike ferrets, guinea pigs do not develop clinical disease upon infection with human or avian influenza viruses [34,35,36]. Avian species are not used to evaluate airborne transmission because the routes of natural infection (i.e., the fecal–oral route in avian species) differ between mammals and birds, and experimental studies indicate that airborne transmission efficiency of avian influenza viruses in domestic chickens is low [37,38].

Both contact and airborne transmission contribute to the spread of an influenza virus in humans [39,40]. In animal models, contact transmission is considered a less stringent indicator of human-to-human transmission [41]; however, all pandemic influenza viruses transmit via contact in ferrets and guinea pigs [30,31,32,33], and this mode of transmission in animal models most likely represents a degree of mammalian adaptation. To evaluate direct contact or airborne transmission, a donor ferret is typically inoculated with a relatively high dose of virus (i.e., 10^6^ infectious units) and contact animals are introduced 24 h later. To study contact transmission, a naïve animal is co-housed in the same cage with the infected donor, while for airborne transmission, the donor and contact animals are placed in a modified cage that allows the animals to share the same air space but prevents direct physical contact. To assess transmission to contact animals, nasal wash samples or swabs are collected and these samples are titrated on Madin–Darby Canine Kidney Epithelial (MDCK) cells. As a secondary method to assess transmission, serological samples from contact animals are also evaluated for seroconversion. Most transmission studies are performed using a limited number of animals (i.e., three or four pairs), thus the assessment of transmission is considered a qualitative measurement [24,26,42].

Based on biochemical characterization and animal studies, several biological changes in a virus are postulated to be required for airborne transmission (extensively reviewed in [43]). These changes include mutations in the polymerase genes to enhance replication in the upper respiratory tract at 33 °C (i.e., PB2 E627K or PB2 D701N) [35,44,45], a change in the HA receptor-binding preference to promote binding to “human-like” α2,6-linked sialic acids (α2,6-SA) with or without a loss of binding to “avian-like” α2,3-linked sialic acids (α2,3-SA) [32,46,47], a decrease in the pH of fusion from 5.7–6.0 to 5.0–5.5 [48,49,50,51,52,53], an increase in the thermostability of the viral HA [54,55], a balancing of neuraminidase (NA) cleavage activity with HA affinity for sialic acids (termed HA–NA balance) [56,57,58], and a change in virion morphology from spherical to filamentous particles [56,59,60]. This paradigm is largely based upon loss-of-function studies with pandemic influenza viruses (i.e., H1N1, H2N2, and H3N2 viruses) [32,43,46,47] and two gain-of-transmission studies with H5N1 viruses [54,55]. Thus, while these changes are considered to be required for airborne transmission of an avian influenza virus, they are not necessarily sufficient and additional properties that have not yet been elucidated are also likely to contribute.

In recent years, influenza viruses of the H5 and H7 subtypes have each caused several hundred human infections with mortality rates exceeding 30%. Severe disease and death are the result of pneumonia and acute respiratory distress syndrome (ARDS). Secondary bacterial infections have been observed in a limited number of patients, but these infections do not appear to play a large role in disease progression and severity [61,62]. As illustrated by the 1918 Spanish influenza pandemic, if an H5 or H7 virus evolves the ability to transmit via the airborne route it could have a devastating disease burden worldwide. Therefore, the goal of this review is to examine the current literature on recent zoonotic H5 and H7 avian influenza viruses with a focus on studies that have characterized transmission in animal models and molecular properties that promote transmission.

## 2. Pathogenicity and Lineages of Avian H5 and H7 Avian Influenza Viruses

Avian influenza viruses are classified as low or highly pathogenic based on their pathogenicity in domestic chickens [63]. Most avian influenza viruses cause a gastrointestinal infection in birds with minimal clinical signs and are classified as low pathogenic. Upon circulation in domestic poultry, viruses of the H5 and H7 subtypes can evolve to become highly pathogenic. Pathogenicity is the result of the accumulation of multiple basic amino acids in the HA cleavage site (termed polybasic cleavage site or polybasic motif) allowing the HA molecule to mature outside of the gastrointestinal tract and establish a systemic infection. Infection of poultry with a highly pathogenic avian influenza virus is characterized by rapid onset and progression of disease associated with high mortality rates [63]. Importantly, some low pathogenic and highly pathogenic H5 and H7 viruses also display virulence in mammals, and some highly pathogenic viruses can cause a systemic infection in animal models [64,65]. Furthermore, the presence of a multiple basic cleavage site is considered a mammalian virulence factor [66], especially when combined with mutations in the viral polymerase that support replication in mammals [67,68,69].

Avian influenza viruses of a specific HA subtype are phylogenetically divided into two main lineages, Eurasian and North American, and these lineages have largely evolved independently due to limited contact between birds on the two continents [15,70,71,72]. Historically, low pathogenic H5 and H7 viruses in both lineages have mutated to become highly pathogenic and have caused outbreaks in poultry [73,74,75,76]. Within the Eurasian lineage, highly pathogenic H5 viruses belonging to the A/goose/Guangdong/1/1996 lineage (GsGd) emerged in the late 1990s and these viruses have caused several hundred zoonotic infections [77,78,79]. Recently, viruses from this lineage have been carried outside Asia by migratory birds and have caused outbreaks in North America. Within the H7 subtype, novel H7N9 viruses emerged in China in 2013 and these viruses have since caused annual epidemics of zoonotic infections [77,80]. Given that both H5 and H7 viruses have displayed the propensity to infect humans, there is heightened concerned that they may evolve the ability to transmit between people and initiate a pandemic.

## 3. Molecular Changes and the Potential for Airborne Transmission of Highly Pathogenic a/Goose/Guangdong/1/1996 Lineage H5 Influenza Viruses

### 3.1. Highly Pathogenic H5N1 Clade 2.1 and 2.2 Viruses

The first reported human infection with highly pathogenic H5N1 was described in Hong Kong in 1997 [81], and the isolated virus belonged to the A/goose/Guangdong/1/1996 lineage (GsGd). In 2003, H5N1 viruses from this lineage again caused human infections, and to date a total of 860 confirmed infections with 454 fatalities have been reported to the WHO [82]. After emerging in China, these viruses have spread to multiple countries and have become endemic in poultry in Bangladesh, China, Egypt, India, Indonesia, and Vietnam [83]. As a result of evolution by drift and reassortment, the GsGd lineage viruses have diversified into 10 clades (0–9) with additional high-order subclades [84]. From 2014 to present, zoonotic infections with highly pathogenic H5N1 have occurred predominantly in Egypt and Indonesia, and these infections have been caused by viruses from clades 2.1 and 2.2, respectively [82,85]. 

In 2012, landmark gain-of-function studies with the highly pathogenic A/Indonesia/5/2005 (H5N1) virus demonstrated that an H5N1 virus could evolve to become airborne transmissible in ferrets via the accumulation of point mutations [54,86]. Specifically, introduction of the mutations HA Q222L and G224S (Q226L and G228S, H3 numbering), and PB2 E627K, followed by 10 serial passages, resulted in the acquisition of additional mutations PB1 H99Y, HA H103Y and T156A (H110Y and T160A, H3 numbering) [87], and airborne transmission between ferrets. The HA mutation H103Y was found to reduce the pH of fusion and increase the temperature stability of the virus, while the T156A mutation enhanced binding to both α2,3 and α2,6-SAs. Introduction of the PB2 E627K mutation increased viral replication and polymerase activity that was further enhanced when combined with the PB1 H99Y mutation [86].

At the same time, gain-of-function studies using a reassortant virus carrying the GsGd lineage H5 HA from A/Vietnam/1203/2004 (H5N1) (Clade 1) combined with the remaining seven gene segments from the 2009 pandemic H1N1 virus were also being performed. In these studies, using a combination of reverse genetics and selection of viral variants, four amino acid changes in the HA N224K, Q226L, N158K, and T318I (H3 numbering) were shown to promote airborne transmission of the reassortant H5N1 virus in ferrets. The HA N224K and Q226L mutations enhanced binding to α2,6-SAs, while the N158K mutation resulted in the loss of a glycosylation site and increased viral replication. Consistent with studies using the A/Indonesia/5/2005 (H5N1) virus, the T318I mutation was shown to decrease the pH of fusion and enhance the thermostability of the virus [55]. Combined, these studies verified that binding to α2,6-SAs and a mammalian adaptation of the viral polymerase are required for airborne transmission, while also identifying the role of reduced pH of fusion and enhanced thermostability of the HA in transmission of avian influenza viruses.

From 2010 to 2015, the majority of human infections with highly pathogenic H5N1 occurred in Egypt, with a total of 256 infections [82]. Since 2016, the number of zoonotic H5N1 infections has decreased to less than 10 per year; however, despite this reduction, highly pathogenic H5N1 viruses remain endemic in domestic poultry in Egypt and other countries. As a result, H5N1 viruses continue to evolve and could potentially acquire mutations that promote transmission to humans. Indeed, phylogenic analysis in 2012 of 46 available H5N1 isolates from Egypt indicated that these viruses lack the HA glycosylation site near residue 156 (158–160 H3 numbering), while also carrying the PB2 E627K mutation [88]. Subsequent, in silico analysis predicated that mutations HA K153D, S223N, and G272S (K157D, S227N, and G275S H3 numbering) that are present in H5N1 viruses circulating in Egypt would result in enhanced binding to α2,6-SAs. When these mutations were introduced into either pseudo-typed retrovirus particles or recombinant influenza viruses they enhanced binding and replication, respectively, to MDCK cells overexpressing α2,6-SAs [89]. Furthermore, evaluation of mutations identified in human isolates from Egypt, including HA H125Y, H125Y/N94D, N182K/T195I/N94D, S223N, and S223N/ S128Δ/I151T (H3 numbering HA H130Y, H230Y/N101D, N186K/T199I/N101D, S227N, and S227N/ S133Δ/I155T), indicated that these mutations enhance binding of recombinant viruses to α2,6-SAs as well as viral replication in primary human airway epithelial cells. However, these mutations also increased the pH of fusion and decreased thermostability of the viruses indicating that the mutations may be destabilizing the HA [90].

#### Ferret Transmission Studies with Recent Highly Pathogenic H5N1 Clade 2.1 and 2.2 Viruses

To determine if the H5N1 viruses that were circulating in Egypt represented an increased pandemic threat, nine poultry viruses isolated between 2014 and 2015 were evaluated for their ability to transmit in ferrets [91]. In initial studies, two viruses, A/duck/Dakahlia/1536CAG/2015 and A/duck/Giza/1529S/2015, transmitted to two of two respiratory contact animals, while the remaining viruses failed to transmit in ferrets. When the transmission studies were repeated, neither the A/duck/Dakahlia/1536CAG/2015 nor the A/duck/Giza/1529S/2015 viruses transmitted to respiratory contact animals in three separate experiments. Using glycan and tissue binding assays, both viruses had a strong binding preference for α2,3-SAs while also exhibiting limited binding to α2,6-SAs [91]. These findings indicate that the viruses were not capable of efficient transmission, but may be able to bind cells in the human upper respiratory tract. Collectively, recent GsGd H5N1 viruses endemic to poultry in Egypt display some propensity to bind α2,6-SAs and this may allow these viruses to establish infections in the upper airways. Still, the viruses have limited ability to transmit via the airborne route, and additional mutations in the HA or reassortment events are likely required for the emergence of a virus that is capable of onwards spread in humans.

### 3.2. Highly Pathogenic H5Nx Clade 2.3.4.4 Viruses

In 2008, highly pathogenic H5 viruses in clade 2.3.4 of the GsGd lineage underwent reassortment in avian hosts. As a result, several NA subtypes including N2, N5, N6, and N8 [92] were introduced and viruses belonging to clade 2.3.4.4 emerged. From this clade, H5N1, H5N6, and H5N8 viruses were carried by migrating birds from China to several countries leading to outbreaks in domestic poultry [93,94,95]. Genetic analysis indicated that viruses within clade 2.3.4.4 evolved into four distinct groups (Groups A–D). Group A consisted of H5N8 viruses that caused outbreaks in China, Korea, Japan, Taiwan, Canada, the United States, and Europe in 2014–2015. Group B H5N8 viruses caused outbreaks in China in 2013 and 2014, and Korea in 2014. Group C included H5N6 and H5N1 viruses in China and Laos in 2013–2014, and H5N1 viruses in China and Vietnam in 2014, respectively. Lastly, Group D consisted of H5N6 viruses that were identified in China and Vietnam in 2013–2014 [84,93,94,95]. In particular, the Group A viruses had a significant economic impact, as they were carried out of Asia down the west coast of Canada and the United States, where they underwent reassortment with North American avian influenza viruses to generate novel H5N1, H5N2, and H5N8 viruses. These viruses were then introduced into commercial poultry farms and spread east across the continent. During these outbreaks H5N2 viruses predominated, and control was finally achieved in the summer of 2015 after 48 million birds were culled at an estimated economic cost of $1.6 billion [95,96,97,98]. In contrast to the parental highly pathogenic GsGd H5N1 viruses, no zoonotic infections were reported during the outbreaks in North America, but have been reported in China with clade 2.3.4.4 viruses of the H5N6 subtype.

#### 3.2.1. Ferret Transmission Studies with Poultry and Environmental Isolates of Highly Pathogenic H5Nx Clade 2.3.4.4 Viruses

As viruses from the GsGd lineage had historically caused zoonotic infections, virus isolates from outbreaks in multiple countries were evaluated for molecular markers indicative of transmission and select isolates were characterized in ferrets. In response to outbreaks of highly pathogenic H5N8 in Korea in January of 2014, the isolate A/mallard duck/Korea/W452/2014 (H5N8) was characterized in human respiratory tissues and ferrets. Relative to a 2009 pandemic H1N1 isolate, the virus grew to 0.5–1 log lower titers in primary human nasal mucosa epithelium, while replicating to comparable titers in human lung tissue explants. The virus maintained a distinct α2,3-SA binding preference, while displaying some binding to α2,6-SAs at high glycan concentrations. In ferrets, both direct contact and respiratory droplet transmission were evaluated. For both modes of transmission, none of the contact animals shed virus; however, two direct contact animals seroconverted indicating low level and inefficient transmission [99]. Subsequent sequence analysis of H5N8 viruses isolated in outbreaks in the Netherlands in 2014 revealed eight amino acid differences between these viruses and viruses isolated in Korea and Japan [100]. In particular, the identification of two mutations, PB2 R699K and HA A185E (A189E H3 numbering), which are located adjacent to position 627 in PB2 and are known to change the antigenicity of H5 viruses, respectively, prompted assessment of the transmissibility of the A/chicken/Netherlands/EMC-3/2014 (H5N8) isolate in ferrets. Following virus inoculation, viral titers declined rapidly in both throat and nasal swabs, and the virus was not transmitted to respiratory droplet contact animals [100]. These findings indicated that the viruses were poorly adapted to mammals and that these mutations did not enhance pandemic potential relative to the Korean viruses.

Following the introduction of H5 viruses to North America, two isolates, A/northern pintail/Washington/40964/2014 (H5N2) and A/gyrofalcon/Washington/41088-6/2014 (H5N8) were evaluated for their ability to replicate in a human bronchial epithelial cell line (Calu-3). When cultured at 33 °C, both viruses grew to reduced (e.g., 10-fold lower) titers relative to a human H3N2 virus, and while the viruses were capable of replicating in inoculated ferrets and caused mild clinical illness, neither virus was capable of direct contact transmission to naïve cage mates [101]. In a separate study, representative Eurasian H5N8 and North American H5N8 and H5N2 viruses were characterized for biological properties of the HA and transmission to direct contact ferrets [102]. All of the viruses exhibited a strong α2,3-SA binding preference with minimal binding to α2, 6-SAs. Fusion was found to occur at pH values of 5.9 or 6.0, and relative to a 2009 pandemic H1N1 isolate, all of the viruses grew to significantly lower titers (>100-fold) in differentiated normal human bronchial epithelial (NHBE) cells. Subsequently, six viruses were evaluated for their ability to replicate in the upper respiratory tract of ferrets, and the viruses replicated poorly with peak titers less than 10^3^ and 10^1^ EID50/mL on days 3 and 5 post-infection, respectively. As in the two previous studies, none of the viruses displayed transmission to direct contact ferrets [99,100,101].

Building upon earlier work on H5 viruses that caused outbreaks in Korea in 2013–2014, two isolates from the 2016–2017 winter season were evaluated for their zoonotic potential [103]. Specifically, A/environment/Korea/W541/2016 (H5N6) and A/common teal/Korea/W555/2017 (H5N8) were studied as representative H5N6 and H5N8 strains. In NHBE cells, the A/environment/Korea/W541/2016 (H5N6) virus grew to significantly higher titers compared to a 2009 pandemic H1N1 strain, while the A/common teal/Korea/W555/2017 (H5N8) virus had reduced titers at 24 h but achieved comparable titers at 48 and 72 h post-infection. In human lung explants, A/environment/Korea/W541/2016 (H5N6) grew to similar titers as the H1N1 virus, while A/common teal/Korea/W555/2017 (H5N8) displayed reduced growth. In ferrets, animals infected with the A/environment/Korea/W541/2016 (H5N6) virus displayed moderate clinical signs and had nasal wash titers of 10^5.3^ and 10^4.8^ EID50/mL on days 3 and 5, respectively. In contrast, A/common teal/Korea/W555/2017 (H5N8) infected animals had minimal clinical illness and had reduced nasal wash titers between 10^2^ and 10^3.8^ EID/mL on day 3 and no viral shedding on day 5. In a separate experiment, infected ferrets were paired with a single direct contact and a single respiratory contact animal. Neither virus transmitted to respiratory contacts, and none of the direct contact animals became infected with A/common teal/Korea/W555/2017 (H5N8); however, three of three direct contact animals shed the A/environment/Korea/W541/2016 (H5N6) virus. Given the propensity for limited transmission, receptor-binding studies were performed and revealed that both viruses had a strong α2,3-SA acid preference, although low-level binding to α2,6-SAs was also observed [103]. In response to case reports of human infections with H5N6 viruses in China, an additional Korean isolate A/mandarin duck/Korea/K16-187-3/2016 (H5N6) was also evaluated in the ferrets. In virus-infected animals, low titers were recovered in the nasal washes (i.e., 10^1.7–2.3^ EID_50_/mL) and the animals did not display overt clinical signs. In transmission studies, neither direct contact or respiratory contact animals shed virus or seroconverted, indicating the virus failed to transmit [104]. Together, these studies demonstrate that the GsGd clade 2.3.4.4 viruses isolated in Korea, North America, and Europe have limited ability to replicate and transmit in mammals.

#### 3.2.2. Zoonotic Infections and Ferret Transmission Studies with Highly Pathogenic GsGd Clade 2.3.4.4 H5N6 Viruses

While the GsGd clade 2.3.4.4 viruses have been widely disseminated and have caused poultry outbreaks in multiple countries, zoonotic infections have only been reported in China. Importantly, these human infections have only been caused by viruses of the H5N6 subtype. Since the first human case in 2014, a total of 19 laboratory confirmed cases of human infections have been reported, with six fatalities [77]. Following reports of human infections, four avian H5N6 isolates that shared high sequence identity with the A/Guangzhou/39715/2014 (H5N6) human isolate were characterized [105,106]. Surprisingly, all four viruses displayed comparable binding to both α2,3 and α2,6-SAs. Two of the viruses were further evaluated in ferrets and infected animals did not display clinical signs; however, both viruses grew to significantly higher titers in upper respiratory tract tissues and grew to comparable or reduced titers in the lungs relative to a highly pathogenic H5N1 isolate. In transmission studies, both H5N6 viruses transmitted within four days to three of three direct contact animals, but failed to transmit to respiratory contact ferrets [105].

In response to increases in the number of reported human infections in 2016, a sequence analysis of nine available human H5N6 isolates was performed [107]. This analysis found that all of the viruses maintained the amino acids 226Q and 228G in the HA (H3 numbering) indicative of an α2,3-SA binding preference, while eight of nine viruses carried mutations that resulted in a loss of the glycosylation site at position 158, suggesting a limited degree of binding α2,6-SAs. In addition, the PB2 E627K mutation was identified in five human isolates, while the PB2 D701N mutation was found in a single virus [107]. Furthermore, all of the H5N6 viruses were found to carry internal genes derived from H5N1, H7N9, or H9N2 viruses that have either caused zoonotic infections or have donated internal genes to viruses that have infected humans [107].

Building upon these observations, detailed studies were performed to characterize the A/Guangzhou/39715/2014 (H5N6) human isolate [108]. This virus displayed a distinct α2,3-SA binding preference and did not bind to α2,6-SAs. The virus also had a higher pH of fusion (i.e., 5.6 vs. 5.3) and reduced thermostability relative to a human H3N2 virus. Surprisingly, at both 33 and 37 °C the A/Guangzhou/39715/2014 (H5N6) isolate exhibited significantly higher polymerase activity compared to a human H1N1 strain and the airborne transmissible A/Indonesia/5/2005 (H5N1) virus. This polymerase activity was shown to be mediated by the PB2 mutation E627K as reversion of the mutation dramatically reduced polymerase activity [108]. In subsequent ferret transmission studies, A/Guangzhou/39715/2014 (H5N6) was shed at high titers up to 10^5^ TCID50/mL in the nasal washes of inoculated donor animals. However, the virus did not transmit to respiratory contact ferrets [108], indicating that despite some degree of mammalian adaptation the virus was not capable of airborne transmission.

### 3.3. Summary of the Potential for Airborne Transmission of Highly Pathogenic GsGd H5 Avian Influenza Viruses

In summary, studies on both the GsGd clade 2.1 and 2.2 virus from Egypt and the clade 2.3.4.4 viruses from multiple countries indicate that these viruses have a limited ability to transmit via the airborne route. While the number of zoonotic infections with clade 2.1 and 2.2 viruses has decreased, these viruses remain a significant threat to human health. The viruses continue to circulate in domestic poultry in the Egypt and could again infect humans in close-contact with these birds. The studies on H5N8 and H5N2 viruses from clade 2.3.4.4 demonstrate that these viruses pose a limited public health risk. While some H5N8 and H5N2 isolates bind to α2,6-SAs, the viruses replicate poorly in mammals and do not transmit between ferrets. In contrast, H5N6 viruses have several characteristics that suggest these viruses will continue to cause zoonotic infections. In particular, some H5N6 viruses display a high degree of binding to α2,6-SAs, in combination with high polymerase activity and the ability to transmit to direct contact ferrets. Based on our current understanding of influenza transmission, it is clear that several of these viruses have attributes that promote airborne transmission. However, the clade 2.3.4.4. H5 HA appears to be relatively unstable with a pH of fusion above 5.5 and low thermostability. Therefore, as with the H5N1 viruses that are endemic in poultry in Egypt, it is likely that additional mutations are required to stabilize the HA molecule and support airborne transmission.

## 4. Molecular Changes and the Potential for Airborne Transmission of Asian Lineage H7N9 Influenza Viruses

### 4.1. Asian Lineage Low Pathogenic H7N9 Influenza Viruses

In the spring of 2013, a novel H7N9 virus emerged in China and began infecting visitors at live-bird markets [109,110]. Phylogenic analysis indicated that the virus emerged through multiple reassortment events in wild birds. Circulating H9N2 viruses donated the internal genes, and separate viruses carrying the H7 and N9 genes provided the HA and NA gene segments. The H7N9 viruses were then introduced into domestic chickens and this species has become the predominant host for these viruses [111,112,113]. Since emerging, the H7N9 viruses have caused five annual epidemic waves of human infections with a total of 1567 cases and 615 deaths [80]. After the first wave of infections during the spring and early summer of 2013, each successive wave started during the winter months (i.e., October–November) and subsided by June. The epidemic wave in 2016–2017 was the most severe with 759 confirmed human cases; this increase in cases most likely resulted from geographical spread of the virus from eastern China throughout the country [114]. Each epidemic wave arose from viruses during the previous wave, and there are currently two separate lineages of H7N9 viruses: the Pearl River Delta lineage and Yangtze River Delta lineage [115,116,117]. Phylogenic analysis of the H7N9 viruses indicates that the NA and internal genes have continued to undergo reassortment with other H7N9 viruses in chickens, while no additional combinations of the H7 HA and different NA subtypes have emerged and caused zoonotic infections [117].

Following the emergence of the H7N9 viruses, multiple research groups rapidly characterized the viruses and assessed transmission in ferrets. Sequence analysis of the early isolates and viruses from subsequent epidemic waves found that the majority of isolates carried the HA mutations G186V and Q226L (H3 numbering), which enhance binding to α2,6-SAs [116,118]. Biochemical characterization of the H7N9 HA revealed that the early viruses displayed an α2,3-SA binding preference, while also exhibiting binding to α2,6-SAs [119,120,121,122,123,124]. Subsequent studies have confirmed that these characteristics are maintained in viruses from the 5th epidemic wave [125,126]. As a result, H7N9 viruses are considered to have dual receptor specificity. Furthermore, the H7 HA was found to lack a glycosylation site at position 158–160 that promoted airborne transmission of the highly pathogenic A/Indonesia/5/2005 (H5N1) virus. Therefore, it is possible that the loss of this site may contribute to the ability of these viruses to infect humans [43].

The vast majority of early isolates were also found to carry mutations in the viral polymerase such as PB2 E627K and D701N [118,127], and these mutations were shown to increase viral replication of H7N9 viruses in mammalian cell lines and animal models [128,129,130]. During the 5th wave of human infections, the frequency of virus isolates carrying the E627K and D701N mutations did not change; however, the number of isolates with PB2 A588V increased to 81%, and earlier studies demonstrated that this mutation combined with PB2 627K increased virulence in mice [127,131]. Collectively, these studies revealed that the H7N9 viruses carried several mutations indicative of the ability to infect and potentially transmit in humans. This was consistent with the observed epidemiology and warranted evaluation of the viruses for their ability to transmit in animal models.

#### Ferret Transmission Studies with Low Pathogenic Asian Lineage H7N9 Viruses

In ferrets, infection with the novel H7N9 viruses isolated in 2013 caused minimal clinical disease and did not result in mortality [132,133,134,135,136,137], although the viruses replicated to high titers in the nose and lungs. In transmission studies, the H7N9 viruses were found to transmit to all direct contact animals [132,137], while different research groups reported variation in proportion of respiratory contacts that became infected. Specifically, the H7N9 viruses were found to transmit to between 33% (i.e., 1 out of 3) to 75 or 100% (i.e., 3/4 or 4/4) of respiratory contact ferrets [132,133,134,135,136,137]. Importantly, the H7N9 viruses displayed delayed transmission kinetics with viral shedding from the respiratory contacts starting on days 3–7 post-exposure, while respiratory contacts exposed to donor ferrets infected with human H1N1 or H3N2 viruses began shedding on days 1–3 post-exposure [132,133].

Several groups performed a sequence analysis of viruses isolated from the respiratory contact ferrets that became infected with the A/Anhui/1/2013 (H7N9) isolate [133,134]. Xu et al. reported transmission to one of four respiratory contact animals, and the virus isolated from the respiratory contact had four mutations: HA R140K (R157K H3 numbering), NA T10I, PB2 D678Y, and NP I109T [135]. The R140K mutation is located on the rim of the receptor-binding pocket and could alter glycan-binding [138]; however, the role of this mutation and the other identified mutations has not been explored. In studies by Watanabe et al., a single respiratory contact animal became infected and the mutations HA T71I, R131K, and A135T (H3 numbering), and NA A27T were identified [134]. The HA mutations R131K and A135T are located in close proximity to the receptor-binding pocket suggesting they may enhance the affinity of the HA for α2,6-SAs, while the T71I mutation is located underneath this pocket indicating it may play a role in stabilizing the HA [134]. In transmission studies by Richard et al., three out of four respiratory contact animals became infected, and a second round of respiratory transmission was initiated using the virus isolated from a respiratory contact that had the fewest substitutions. In this second round of transmission, only one in four respiratory contacts became infected [133], yet all of the respiratory contacts in both rounds of transmission had the HA mutations N123D (N133D H3 numbering) and N149D (located between amino acids 158 and 159 in H3 numbering). These mutations are also located adjacent to the receptor-binding site, but do not interact directly with sialic acids. When the role of these mutations was examined using recombinant viruses, introduction of the N123D and N149D mutations resulted in a modest 2–4-fold increase in binding to α2,6-SAs [133]. Further evaluation of the pH of fusion demonstrated that the HA of A/Anhui/1/2013 (H7N9) underwent fusion at pH 5.6 and neither the N123D or N149D mutations modified this property. In two respiratory contacts, the mutation PB1 M523I was also identified, although this mutation did not alter polymerase activity at 33 or 37 °C. In addition to the HA N123D and N149D mutations, deep sequence analysis identified the receptor binding variant HA L217Q (L226Q H3 numbering) at relatively low frequencies (i.e., ~9–24.5%) in three donor ferrets. This mutation was not present in the respiratory contact animals, but was later shown to decrease the pH of fusion from 5.6 to 5.4, while also increasing the temperature stability of the virus [139]. The role of the HA G219S (G228S H3 numbering) mutation, which is known to promote binding to α2,6-SAs in H3 and H2 influenza viruses and enhance binding of H7N9 viruses to human trachea and alveolar epithelium, was also evaluated. This mutation was found to increase the pH of fusion to 6.0 and decrease the temperature stability of the virus [139]. Jointly, these studies indicate that the H7N9 HA is not optimally stabilized for airborne transmission, and the introduction of mutations known to enhance glycan binding or transmission may not readily stabilize the HA.

To evaluate if viruses from the second and third epidemic waves exhibited enhanced transmission, four second-wave and two third-wave viruses were compared [140]. The majority of viruses carried the PB2 627K and HA 217Q (226Q H3 numbering) mutations, and all of the viruses replicated in human bronchial epithelial cells at 37 °C, although there were strain specific differences. When viral replication was evaluated at 33 °C, all of the viruses displayed reduced replication at 12 and 24 h post-infection but reached similar titers to those obtained at 37 °C by 48 h. These findings indicated that the viruses have not yet acquired the ability to efficiently replicate at 33 °C. Using a virus-induced hemolysis assay as a surrogate for pH of fusion, the A/Anhui/1/2013 virus and all of the second epidemic wave viruses displayed fusion at pH 5.8, while the third wave viruses exhibited a pH of fusion of 5.6. These data suggests that the third wave viruses may be adapting to mammals. Representative second- and third-wave viruses, A/Hong Kong/5942/2013 and A/British Columbia/1/2015, respectively, were then evaluated for direct and respiratory contact transmission in ferrets. Both viruses transmitted to three out of three direct contacts and one out of three respiratory contacts. The transmission kinetics and proportion of animals that became infected were similar to that of A/Anhui/1/2013 (H7N9), and all of the viruses exhibited delayed transmission to the respiratory contacts [140]. These studies indicate that the H7N9 viruses are not capable of efficient respiratory transmission, and consistent with these findings, the H7N9 viruses have not shown evidence of enhanced or sustained human-to-human transmission over the five epidemic waves [141].

Importantly, gain-of-transmission studies and molecular characterization of mammalian adaptive mutations have been performed with other H7 virus strains. These strains include a highly pathogenic H7N1 isolate and two H7N7 viruses. While these strains belong to the Eurasian lineage of H7 viruses, they are distantly related to the Asian lineage H7N9 viruses [142,143,144]. In these studies, after 10 serial passages of a highly pathogenic H7N1 virus carrying the PB2 E627K mutation, five amino acid mutations were enriched and the virus exhibited the ability to transmit to two out of three respiratory contact ferrets. One of the mutations was in the stalk region of the HA (K/R304R H3 numbering, K/R295R, H7 numbering) suggesting a potential role in stabilizing the HA, while the remaining mutations were in the PB2, NP, and M genes. The location of these later mutations suggested that they may enhance polymerase activity or modify the stability of the virion [144]. Similarly, evaluation of mutations present in highly pathogenic H7N7 viruses that caused human infections in 2003, found that mutations in PA and NA contributed to efficient replication in human cells and virulence in mice [142], and mutations in the viral polymerase of a mouse adapted avian-origin H7N7 virus were found to be the determinants of adaptation and virulence in mice [143]. These studies suggest that H7 viruses may adapt to mammals and evolve the ability to transmit via the airborne route through previously undefined or understudied mechanisms. Therefore, future studies should focus on both genetic signatures of transmission and biological characterization of emerging H7N9 viruses.

### 4.2. Asian Lineage Highly Pathogenic H7N9 Influenza Viruses

The first human infection with a highly pathogenic variant of H7N9 was reported in December of 2016 [145,146] during the 5th epidemic wave. Sequence analysis indicated that HA cleavage site contained a polybasic motif, indicating a highly pathogenic phenotype in poultry. From December 2016 until September 2017, a total of 32 human infections with highly pathogenic H7N9 were reported [147,148,149], and all of the highly pathogenic variants belong to the Yangtze River Delta lineage [117]. Highly pathogenic H7N9 viruses were also isolated from chickens, suggesting the virus originated in these animals [116,117,150,151].

Analysis of available genome sequences for 28 human and 21 environmental isolates of highly pathogenic H7N9 found that 25/28 human and all of the environmental samples had a reversion of the HA mutation at position 226 from leucine (L) to glutamine (Q) (H3 numbering) [117]. All of these isolates maintained the HA mutation G186V (H3 numbering) that is associated with enhanced binding to α2,6-SAs. In the viral polymerase, 14 and 4 of the human isolates had the substitutions PB2 E627K and D701N, respectively [117], while none of the environmental isolates carried these mutations. The reversion of the HA L226Q mutation was surprising as the 226L mutation was previously shown to enhance binding to α2,6-SAs [139]. When receptor-binding preference of the highly pathogenic human isolate A/Guangdong/17SF003/2016 (H7N9) was evaluated using glycan arrays, the virus displayed an α2,3-SAs binding preference. In contrast, when binding preference was assayed using biolayer interferometry, binding to both α2,3 and α2,6-SAs was observed [152], and this was consistent with receptor-binding studies on the A/Guangdong/17SF003/2016 and A/Guangdong/17SF006/2017 (H7N9) isolates using an ELISA-based assay [126]. Together, these studies indicate that the highly pathogenic H7N9 viruses retain the dual receptor-specificity observed with the low pathogenic precursor viruses, although it is unclear if reversion of the HA L226Q mutation reduced or altered the affinity of the highly pathogenic HA for α2,6-SAs. Importantly, as noted above, reversion of this mutation in the low pathogenic A/Anhui/1/2013 (H7N9) virus reduced the pH of fusion [139], and it is possible that this mutation may have been selected to stabilize the HA.

#### Ferret Transmission Studies with Highly Pathogenic Asian Lineage H7N9 Viruses

To date, one human and one chicken isolate of highly pathogenic H7N9 have been evaluated for transmissibility in ferrets [151,152]. In studies by Shi et al., the highly pathogenic poultry isolate A/chicken/Guangdong/SD008/2017 (H7N9) [CK/SD008] was compared to the low pathogenic A/Anhui/1/2013 (H7N9) virus. Both viruses were found to bind α2,3-SA and α2,6-SAs, and the CK/SD008 virus displayed slightly enhanced thermostability relative to A/Anhui/1/2013. In respiratory transmission studies, the CK/SD008 virus transmitted to one out of four respiratory contact ferrets. By performing limiting dilutions on viruses recovered from infected ferret lung tissues, Shi et al. isolated variants carrying the PB2 627K or 701N mutations. When these polymerase variant viruses were used to initiate airborne transmission studies, the PB2 627K-CK/SD008 virus transmitted to 100% of the respiratory contact animals, while the PB2 701N-CK/SD008 virus transmitted to 66% of contacts. These studies suggest that the highly pathogenic CK/SD008 virus has the potential to readily adapt to mammals and may be capable of on-ward transmission. However, while the polymerase variants displayed enhanced transmission relative to the wild-type CK/SD008 virus, when transmission of the low pathogenic A/Anhui/1/2013 (H7N9) virus was evaluated in parallel, 100% of the respiratory contacts also became infected. Given that this is one of the few studies to report transmission of the A/Anhui/1/2013 (H7N9) virus to 100% of the respiratory contacts, it is difficult to determine if the highly pathogenic viruses carrying polymerase mutations have enhanced potential relative to the low pathogenic strains to transmit via the airborne route [151].

Imai et al. studied the human isolate A/Guangdong/17SF003/2016 (GD/3) and performed a deep sequencing analysis of variant populations [152]. This analysis identified a subpopulation of variants that contained the mutation NA R294K, a known neuraminidase inhibitor resistance marker. As a result, the wild-type egg-passaged virus, and two recombinant viruses carrying either the NA 294R or 294K mutations on the GD/3 backbone were generated [152]. In differentiated NHBE cells, the recombinant GD/3 virus carrying N294R (rGD/3 NA 294R) grew to comparable titers to the low pathogenic A/Anhui/1/2013 (H7N9) virus at both 33 and 37 °C, while the wild-type virus and GD/3 NA 294K virus displayed reduced growth at 33 °C. In contrast, at 37 °C, only the rGD/3 NA 294K virus displayed reduced growth kinetics demonstrating that this mutation attenuated the virus. Similarly, in ferrets on day 3 post-infection, all three highly pathogenic viruses and the low pathogenic A/Anhui/1/2013 virus replicated efficiently in the nasal turbinates and lungs, although the rGD/3 NA 294K virus replicated to significantly lower titers in the lungs. On day 6 post-infection, all of the highly pathogenic viruses grew to comparable titers in the lungs and nasal turbinates, and replication of the low pathogenic A/Anhui/1/2013 (H7N9) virus was reduced in the lungs.

In transmission studies, mortality was observed in donor ferrets inoculated with the highly pathogenic wild-type and recombinant GD/3 H7N9 viruses, while none of the A/Anhui/1/2013 (H7N9) infected ferrets became severely ill. One GD/3, two rGD/3 NA294R, and one rGD/3 NA 294K infected donor animals died between days 4 and 8 post-infection [152]. Transmission occurred by respiratory droplet to one, three, two, and one out of four exposed animals for GD/3, rGD/3 NA 294R, rGD/3 NA 294K, and A/Anhui/1/2013 (H7N9) viruses, respectively. For respiratory contacts exposed to the rGD/3 NA 294R, two out of three animals that became infected developed severe disease and succumbed to infection on days 6 and 9 post-exposure, and the respective donor ferrets for these animals also succumbed to infection (as noted above) [152]. These studies indicate that the highly pathogenic H7N9 viruses have enhanced virulence in ferrets, while respiratory transmission is inefficient and exhibits a similar degree of variation as reported for the low pathogenic A/Anhui/1/2013 (H7N9) virus [132,133,134,135,136,137]. Therefore, it is likely that as with the low pathogenic H7N9 strains, highly pathogenic variants will also require mutations to stabilize the HA. Given that previously identified mutations do not readily confer stability to the H7 HA, novel mutations or drift variants identified in both highly pathogenic and low pathogenic H7N9 viruses should be rapidly characterized for their effect on pH of fusion, thermostability, and airborne transmission in ferrets.

### 4.3. Summary of the Potential for Airborne Transmission of Asian Lineage Low and Highly Pathogenic H7N9 Viruses

Taking into account the epidemiological findings over the five epidemic waves of human infections and biological characterization of both the low pathogenic and highly pathogenic strains of H7N9, the Asian lineage of H7N9 viruses, are recognized by the CDC as the influenza viruses with the highest potential pandemic risk [153]. These viruses display variation in their ability to transit between ferrets with different investigators reporting airborne transmission to 33–100% of contact animals. Based on studies that characterize the HA of the H7N9 viruses, instability of the HA in both low and highly pathogenic H7N9 viruses is likely inhibiting or limiting transmission via the airborne route. While there have been five waves of human infections and highly pathogenic H7N9 variants have emerged, during the expected 6th epidemic wave from October of 2017 to July of 2018, only a single human case of H7N9 infection was reported. The reasons for this decrease in zoonotic infections are unclear; however, the Chinese government initiated a large H7N9 and H5 vaccination campaign in poultry [154] and this may be limiting the spread of the disease. Despite this campaign, outbreaks of HPAI H7N9 in poultry are ongoing [155], and the potential for future outbreaks and zoonotic infections remains. Importantly, vaccination of poultry does not typically confer sterilizing immunity, and most avian influenza vaccines reduced clinical illness and spread but do not block infection or viral replication in birds [156,157]. As a result, vaccination may drive antigenic drift of the virus as observed Marek’s disease [158], and this may result in the reemergence of the H7N9 viruses. Therefore, it is now increasingly important to monitor viral evolution with a focus on changes that may stabilize the viral HA, and perform pandemic risk assessment on viruses isolated during poultry outbreaks and from zoonotic infections.

## 5. Conclusions

Highly pathogenic GsGd H5 viruses and Asian lineage H7N9 viruses pose a significant pandemic threat. While the number of zoonotic infections with these viruses has decreased in the past year, H5 viruses remain endemic in poultry in the Middle East, and H7N9 viruses continue to circulate and cause poultry outbreaks in China. Both H5 and H7 viruses carry several molecular markers indicative of the ability to infect and transmit in humans, although the HA molecule of these viruses is relatively unstable and this likely represents the most significant obstacle to emergence of a virus capable of efficient airborne transmission. As the initiation of a pandemic requires the optimal balance of viral properties to be combined in a single virus, the presence of viruses circulating in nature with several of these changes that are also capable of causing severe disease in humans warrants significant pandemic preparedness efforts. Indeed, the 1918 Spanish influenza pandemic is a stark reminder of the potential for influenza viruses to impart a devastating global disease burden. Surveillance efforts and laboratory characterization are fundamental to assess the threat posed by emerging H5 and H7 viruses, and significant emphasis should be focused on understanding the mutations or changes that would stabilize the HA. On-going research is needed to develop pandemic influenza vaccines, and combined with basic research, both approaches could enhance our ability to predict and potentially reduce the burden of an H5 or H7 influenza virus pandemic.
